# Sustainable extraction of polyphenols from *Pleurotus eryngii* using ultrasound-assisted deep eutectic solvents with process optimization and biological activity^☆^

**DOI:** 10.1016/j.ultsonch.2026.107937

**Published:** 2026-06-28

**Authors:** Muhammad Waheed Iqbal, Ping Shao, Yang Lin, Sanabil Yaqoob, Muhammad Shoaib, Qing Shen, Moneera O. Aljobair, Isam A. Mohamed Ahmed

**Affiliations:** a State Key Laboratory of Green Chemical Synthesis and Conversion, Zhejiang University of Technology, Zhejiang, Hangzhou, 310014, China; b College of Food Science and Technology, Zhejiang University of Technology, Zhejiang, Hangzhou 310014, China; c Moganshan Research Institute at Deqing County Zhejiang University of Technology, Huzhou 313200, China; d Laboratory of Food Nutrition and Clinical Research, Institute of Seafood, Zhejiang Gongshang University, Hangzhou, 310012, China; e College of Biosystems Engineering and Food Science, Zhejiang University, Hangzhou, 310058, China; f Department of Food Sciences and Nutrition, College of Food and Agricultural Sciences, King Saud University, P. O. Box 2460, Riyadh 11451, Saudi Arabia; g Department of Sports Health, College of Sports Sciences and Physical Activity, Princess Nourah bint Abdulrahman University, Riyadh, Saudi Arabia

**Keywords:** *Pleurotus eryngii*, Polyphenols, Deep eutectic solvent, Ultrasonic-assisted extraction, Antioxidant and antifungal activity

## Abstract

Ultrasound-assisted extraction using a deep eutectic solvent (UDES) represents sustainable strategy for recovering polyphenols from *Pleurotus eryngii* (PEPs). In this study, preliminary single-factor experiments were first conducted to evaluate the effect of individual extraction parameters. The conditions were subsequently optimized through response surface methodology (RSM), resulting in optimal parameters of 50 °C temperature, 320 W ultrasonic power, 60  g/L solid-to-solvent ratio, and 20 min extraction time, with a predicted PEPs yield of 39.60  mg GAE/g and an actual yield of 37.03 ± 1.3  mg GAE/g. Extraction kinetics were well described by a pseudo-second-order model (R^2^ > 0.99), while FTIR spectroscopy confirmed the integrity of key functional groups in the optimized DES components and verified the successful ultrasound-assisted extraction of polyphenols using different methods. Similarly, SEM analysis revealed clear morphological changes between the raw sample and the powder extracted using water, ethanol and DES both with and without ultrasonication highlighting clear structural changes during extraction process. HPLC identified vanillic acid (9.14 mg/g) and gallic acid (7.46 mg/g) as the main phenolic compounds. Furthermore, this study identified fumaric acid, 6,7-dihydroxycoumarin, and caffeic acid in *Pleurotus eryngii* for the first time, expanding its known polyphenolic profile. UDES-extracted PEPs showed greatly enhanced antioxidant activity across ABTS, DPPH, and FRAP assays and demonstrated strong antifungal effects, inhibiting Aspergillus niger growth by 86.6% at 0.35 mg/mL. Overall, these findings demonstrate that UDES is a high-efficient, sustainable approach for extracting bioactive polyphenols from *P. eryngii.* The results also highlight its potential for the green valorization of mushroom by-products and their application in functional food as well as nutritional values in food system.

## Graphical abstract

**Figure:**
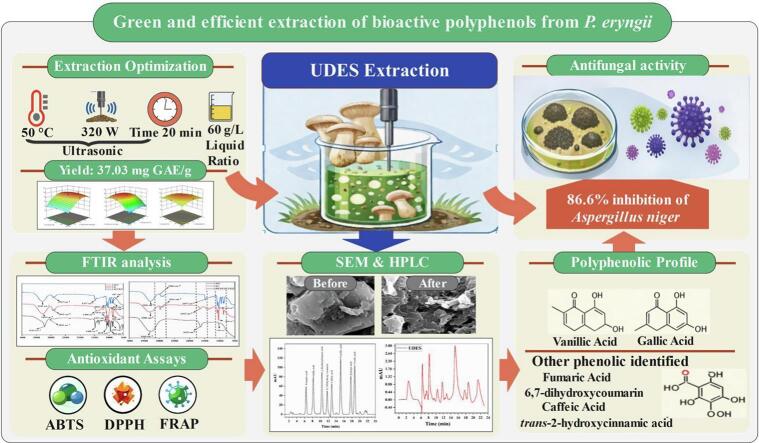

## 1 Introduction

Mushrooms have been cherished by many people for centuries because of their distinctive texture, aroma, and taste. Recent studies have demonstrated the wide range of nutrients and bioactive compounds produce various health benefits from eating mushrooms [1]. Over the past few decades, growing consumer demand for mushrooms has led to a 30-fold increase in global mushroom production, with more than 40% of the total production coming from the *Lentinus edodes* and the *Pleurotus* (oyster mushroom) genera [2]. *Pleurotus eryngii* (commonly known as the king trumpet or king oyster mushroom) is one of the most commercially valuable edible mushrooms due its high nutritional value and rich bioactive composition. It contains abundant nutritional components include large quantities of protein, polysaccharides, dietary fiber, vitamins, minerals and other bioactive compounds, making it an excellent source of low-fat functional foods [3], [4]. The fruiting body of *P. eryngii* are rich in nutrients and contain high levels of phenolic acids present, which significantly contribute to their antioxidant activity and preventing oxidative stress [5].

The processing of *P. eryngii* generates a substantial number of by-products, including mushroom bases, irregular stalk portions, trimming residues, and damaged fruiting bodies that are usually discarded during industrial processing and commercialization. These by-products also contain valuable bioactive compounds, particularly polyphenols, and therefore represent a promising low-cost resource for valorization [6]. These fungal-derived phenolic compounds have received increasing attention for their wide variety of potential health benefits such as antioxidant, anti-inflammatory, and antimicrobial activity and are used in functional foods and nutraceuticals consumed in everyday diets [7]. However, phenolic compounds in fungi have often been overlooked versus those found in higher plants [8]. A primary reason for limited research on fungal phenolic acids compared with plants is the complex fungal cell wall structure. As the fungal cell walls contains substantial amount of chitin, *β*-glucans, and a tightly cross-linked polysaccharide, which bind phenolic acids and reduce their accessibility and extractability [9]. Furthermore, fungi relatively contains lower phenolic concentrations than plants, and the limited number of optimized extraction methods, further hinder the efficient recovery of phenolic acids [10]. Most importantly, byproducts and waste generated from mushroom cultivation such as residual biomass, are a rich source of phenolic compounds that remains largely underutilized [11]. Consequently, developing effective extraction methods to disrupt cell wall barriers and liberate both bound and free phenolics is essential for sustainable valorization of mushroom industrial waste and recovery of high-value bioactive compounds.

The efficient extraction of fungal polyphenols mainly based on the extraction methods and solvents employed. Conventional extraction methods using water and other organic solvents are often inefficient and time consuming. They may also require high temperatures, or toxic solvents, which can degrade heat sensitive phenolic compounds [12]. Due to these limitations, in line with European Union environmental policies (2010–2050) focused on reducing the use of petrochemical solvents and volatile organic compounds [13]. Many countries have turned their attention towards finding eco-friendly alternatives called green extraction methods. Among these approaches, deep eutectic solvents (DESs) have emerged as promising green extraction solvents for fungal phenolics due to their adjustable polarity and high solvation capacity, low toxicity and relatively low costs. They are characterized by strong hydrogen bonding networks formed through interactions between hydrogen bond acceptors (HBA) and hydrogen bond donors (HBD). In addition, by changing the type and molar ratio of HBA and HBD can alter the polarity as well as viscosity of DES, enabling the selective extraction of target metabolites from a fungal source [14]. Similarly, ultrasonic-assisted extraction (UAE) is an efficient technique whereby alternating compression and rarefaction of a liquid forms cavitation bubbles that collapse violently, producing localized high temperatures, pressures and shear forces. The mechanical effects of acoustic cavitation can disrupt fungal cell walls, thereby enhancing solvent penetration as well as mass transfer and increases the polyphenols recovery from the mushroom matrix [15], [16], [17]. However, studies investigating the use of ultrasound-assisted deep eutectic solvents (UDES) for phenolic acids extraction from fungi remains limited.

Despite growing interest in DESs for the extraction of bioactive compounds, limited information is currently available on using UDES for recovering polyphenols from fungal biomass, particularly from *P. eryngii* production waste (PEPs). To address this gap, the present study applied an optimized UDES extraction approach, using preliminary single-factor experiments followed by response surface methodology (RSM) to determine the optimal extraction conditions of PEPs. The functional properties of the extracted PEPs, including their antioxidant and antifungal activities were subsequently evaluated, which to the best of our knowledge have not been previously reported for this system. In addition, kinetic modeling was used to characterized the extraction process, while FTIR and SEM analysis were conducted to elucidate the synergistic effects of ultrasonication during extraction. Overall, the findings provide a new green and efficient strategy for recovering PEPs from mushroom by-products and demonstrate the advantages of UDES over conventional extraction methods.

## 2 Materials and methods

### 2.1 Materials

Fresh *P. eryngii* processing by-products were obtained from Nanjing Chunquan Food Co., Ltd. (Fujian, China). The by-products mainly contained of mushroom bases, irregular stalk portions, trimming residues, as well as damaged fruiting body tissues excluded from commercial sale and consumption. The collected material samples were cleaned, sliced, freeze-dried, and finely ground using a high-speed grinder, then sieve through a 40-mesh screen. The prepared powder was preserved at –20 °C in airtight containers until further use.

The following analytical grade chemicals were purchased from commercial suppliers and used without further purification: choline chloride (ChCl, ≥98%), glycerol (Gly, ≥99%), lactic acid (LA ≥ 98%), citric acid (CA, ≥99%), fructose (Fruc, ≥99%), ethylene glycol (EG, ≥99%), urea (U, ≥99%), betaine (Be, ≥98%), malic acid (MA, ≥99%), 1,4-butanediol (But, ≥99%), glucose (Glu, ≥99.5%), sodium carbonate (≥99.8%), and potassium persulfate (K_2_S_2_O_8_, ≥99.5%). All chemicals were sourced from Aladdin Reagent Co., Ltd. (Shanghai, China) and Macklin Biochemical Technology Co., Ltd. (Shanghai, China). HPLC grade standards such as fumaric acid (≥99%), gallic acid (≥99%), *p*-hydroxybenzoic acid (≥99%), 6,7-dihydroxycoumarin (≥98%), caffeic acid (≥99%), vanillic acid (≥99%), syringic acid (≥98%), and ferulic acid (≥99%), were obtained from Sigma-Aldrich GmbH (Steinheim, Germany) and Aladdin Reagent Co., Ltd. (Shanghai, China). The reagents used for antioxidant assays 2,2′-azino-bis (3-ethylbenzothiazoline-6-sulfonic acid) diammonium salt (ABTS, ≥98%), 2, 2-diphenyl-1-picrylhydrazyl (DPPH, ≥98%), and 6-hydroxy-2,5,7,8-tetramethylchromane-2-carboxylic acid (Trolox, ≥98%) were purchased from Sigma-Aldrich Co. LLC. (St. Louis, MO, USA). The 2,4,6-Tris(2-pyridyl)-s-triazine (TPTZ, ≥99%), iron (III) chloride hexahydrate (FeCl_3_·6H_2_O, ≥99%), and sodium acetate (≥99%) were procured from Aladdin Reagent Co., Ltd. (Shanghai, China) and Shanghai Maclin Biochemical Technology Co., Ltd. (Shanghai, China). The potato dextrose agar (PDA) and potato dextrose broth (PDB) were obtained from Hope Bio-Technology Co., Ltd. (Qingdao, China). All other chemicals used in the buffer preparations were of analytical grade unless stated otherwise.

### 2.2 Preparation of DESs

A total of 12-DESs were synthesized following the method reported by Sun et al. [18], with slight modifications (Table 1). The HBAs were mixed with various HBDs according to the established molar ratios. Each mixture was prepared in a conical flask equipped with a magnetic stirring bar and heated at 80 °C with constant stirring until a clear, stable, and homogeneous viscous liquid was obtained. The prepared DESs were kept at room temperature for 12  h to ensure complete formation process. The preparation was deemed successful when no crystallization or phase separation was observed. For the purpose of viscosity reduction, and increase solvent polarity, selected DESs were subsequently diluted with 10–20% (v/v) distilled water and stored in airtight containers at room temperature until further use.

**Table 1:** Composition and physicochemical properties of the synthesized DESs, including HBA and HBD.

### 2.3 Physicochemical characterization of the DESs

The viscosity of synthesized DESs was determined using a rotational viscometer (Model SNB-1; Shanghai Jitai Electronic Technology Co., Ltd. Shanghai, China) at 25,35,45,55, and 65 °C under the fixed shear rate of 0.1  s^–1^ for a duration of 10  s.

The density of DESs was determined using the standard density bottle method. DES samples were added into a pre-weighed density bottle and maintained at a constant temperature for 30 min. The density (ρ) values were obtained using equation (1):

(1) $$\rho =\frac{m_{1}-m}{m_{2}-m}\times 0.9982$$

where *m_1_* is the mass of the bottle filled with DES (g), *m* is the mass of the empty density bottle (g), *m_2_* is the mass of the bottle filled with distilled water (g), and 0.9982  g/cm^3^ is the density of water at room temperature.

The pH of each synthesized DES was determined using a calibrated PHS-25 pH meter (Shanghai Jiepeng Scientific Instruments Co., Ltd., Shanghai, China) at 20, 30, 40, 50, 60, and 70 °C after equilibrating the samples for 5 min at the target temperature.

The hydrogen bond formation in the optimized DES composed of ChCl as a HBA and EG as a HBD at a molar ratio of 1:2, was accessed by a Nicolet 6700 FTIR spectrometer (Thermo Fisher Scientific, USA). The spectrum of the FTIR was recorded in the range of 500–4000  cm^–1^. Characteristic shifts and broadening in the O-H and N-H stretching vibrations were used to verify strong hydrogen bonding interactions between ChCl and EG, indicating the successful and stable synthesis of the DES.

### 2.4 Extraction of PEPs

The extraction was carried out using all 12 kinds of DES assisted UAE extraction process at a solid-to-solvent ratio of 50 g/L in a closed conical flask with a magnetic stirring bar. During PEPs extractions, the samples were subjected to ultrasound treatment under controlled ultrasonic power conditions while maintaining continuous stirring in a thermostatic water bath at 60 °C for 30 min. The extraction parameter ranges applied in this study were chosen based on preliminary experiments. Water and ethanol were used as comparative solvents. After extraction, the samples were cooled to room temperature, centrifuged at 8000 rpm for 15 min, and the supernatants were collected for analysis. For comparison control extractions were performed without UAE using water (WC), ethanol (EC), or the optimized DES (ChCl-EG), and with UAE (UWC, UEC, and UDES/ChCl-EG).

### 2.5 Quantification of PEPs content

The total phenolic content (TPC) of PEP extracts was measured with slight modifications to the method of Mehmood et al. [19]. In brief, 25  µL of mushroom extract or gallic acid (GA) standard was combined with 125  µL of 10-fold diluted Folin-Ciocalteu reagent in a 96-well microplate and incubated for 1  min. Subsequently, 100  µL of sodium carbonate solution (75  mg/L) was added in it, and the reaction mixture was incubated at room temperature for 30  min. Absorbance was recorded at 765  nm (Tecan Spark, Männedorf, Switzerland), and TPC was expressed as mg GAE/g dry weight (DW).

### 2.6 Optimization of PEPs extraction efficiency

#### 2.6.1 Effect of different extractions conditions on the yield of PEPs

UAE parameters were optimized by evaluating the effects of solid-to-solvent ratio, extraction temperature, extraction time, and ultrasonic power on polyphenol yield. For all experiments, *P. eryngii* powder was dispersed in the optimized DES (ChCl-EG, 1:2) containing 10% water, and the extraction yield was assessed based on TPC. The optimal solid-to-solvent ratio was assessed at concentrations of 30, 40, 50, 60, 70, and 80 g/L under conditions of 240 W, 60 °C, for 30 min. The effect of temperature was then evaluated at 30, 40, 50, 60, and 70 °C using a fixed solid-to-solvent ratio of 60 g/L, ultrasonic power of 240 W, and extraction time of 30 min. For extraction time optimization, UAE was conducted at 240 W and 50 °C for 10, 20, 30, 40, 50, and 60 min. Finally, the influence of ultrasonic power was examined by setting the output to 240, 280, 320, 360, and 400 W under conditions of 60 g/L solid-to-solvent ratio and 50 °C for 20 min.

#### 2.6.2 Optimization of PEPs extraction using RSM

Based on the results of the single-factor experiments, four key factors, ultrasonication time (A, min), ultrasonication temperature (B, °C), solid-to-solvent ratio (C, g/L), and ultrasonication power (D, W) were selected as independent variables for optimization of PEPs extraction. RSM with a Central Composite Design (CCD) was applied using Design Expert-13 software to evaluate the combined effects of these factors at three levels (low, medium, high), coded as − 1, 0, and **+** 1 are given in Table 2. The extraction yield of total PEPs (Y, mg GAE/g) was used as the dependent variable. A total of 33 experiments were conducted according to the CCD, including replicates at the central point, and the resulting data were fitted to a quadratic (second-order) polynomial equation model to identify the optimal extraction conditions (Table 3).

**Table 2:** Independent variables and corresponding coded levels used for TPC determination.

**Independent variable**	**symbols**	**Coded levels**				
		**−α**	**−1**	**0**	**+1**	**+α**
Time (min)	X_1_	0	20	40	60	0
Temperature (°C)	X_2_	0	30	50	70	0
Solid to solvent ratio (g/L)	X_3_	0	30	50	70	0
US power (W)	X_4_	0	240	320	400	0

**Table 3:** Experimental and predicted values of TPC.

Independent variables					Response Values TPC (mg GAE/g)	
Run	**Time (min)**	**Temperature (°C)**	**Solid to solvent ratio (g/L)**	**Ultrasonic power (W)**	**Experimental values**	**Predicted values**
1	20	30	30	240	21.6025	21.23
2	60	30	30	240	22.0068	21.56
3	20	70	30	240	22.4028	22.89
4	60	70	30	240	22.7505	23.22
5	20	30	70	240	32.6553	33.31
6	60	30	70	240	30.8455	31.70
7	20	70	70	240	34.8147	34.85
8	60	70	70	240	33.6673	33.24
9	20	30	30	400	22.2728	22.65
10	60	30	30	400	23.2371	23.47
11	20	70	30	400	22.6419	22.05
12	60	70	30	400	23.5791	22.87
13	20	30	70	400	35.4511	35.25
14	60	30	70	400	34.6604	34.12
15	20	70	70	400	34.1437	34.54
16	60	70	70	400	32.7781	33.41
17	20	50	50	320	39.5572	38.76
18	60	50	50	320	38.4178	38.36
19	40	30	50	320	35.6589	35.09
20	40	70	50	320	35.8444	35.56
21	40	50	30	320	25.5301	26.08
22	40	50	70	320	38.8067	37.40
23	40	50	50	240	37.1041	35.85
24	40	50	50	400	36.2461	36.64
25	40	50	50	320	37.4775	37.91
26	40	50	50	320	37.5284	37.91
27	40	50	50	320	38.3486	37.91
28	40	50	50	320	36.7054	37.91
29	40	50	50	320	38.2018	37.91
30	40	50	50	320	37.7054	37.91
31	40	50	50	320	38.1751	37.91
32	40	50	50	320	37.5954	37.91
33	40	50	50	320	36.8578	37.91

#### 2.6.3 Validation of optimal process conditions

The TPC of samples prepared under the predicted optimal conditions was determined experimentally. The obtained results were then compared with the model-predicted values to evaluate the accuracy of the RSM model and verify the suitability of the optimized conditions for maximum polyphenol recovery (Table 3).

### 2.7 Extraction kinetics

The extraction kinetics of PEPs were evaluated using a second-order kinetic model, as described by Lazar et al. [20]. Dynamic fitting was carried out under optimized conditions (ultrasonication time: 0–27 min, power: 351 W, temperature: 49.23 °C, and solid-to-solvent ratio: 58 g/L). The TPC of samples collected at defined time intervals was determined.

The extraction of PEPs by UDES can be represented by equation (2):

(2) $$dC_{t}/dt=k{\left(C_{s}-C_{t}\right)}^{2}$$

where *C_t_* denotes the TPC (mg GAE/g) at time t (min), *C_s_* denotes the equilibrium TPC concentration (mg GAE/g), and *k* is the second-order extraction rate constant (g·min^–1^·mg^–1^). By linear transformation of equation (2), the model can be expressed as equation (3):

(3) $$\frac{t}{C_{t}}=\frac{1}{k}.C_{s}^{2}+t/C_{s}={\frac{1}{h}+t/C}_{s}$$

As *C_t_* and *t* approach 0, *h* is defined as the initial extraction rate (g^–1^·min^–1^). Equation (3) was reorganized to obtain (4) where *C_t_* represent the TPC at any given time.

(4) $$C_{t}={t/\left(\frac{1}{h}\right)+(\frac{t}{C}}_{s})$$

the initial extraction rate (*h*), the solvent saturation TPC (*C_S_*), and the secondary extraction rate constant (*k*) were obtained from the slope and intercept of the linearized relationship form of equation (3) (Table 4).

**Table 4:** Second-order kinetic parameters for PEPs extraction under different conditions.

**Extraction method**	***C_s_* (mg/g)**	***h* (mg/min g)**	***k* (g⋅/min mg)**	**R^2^**
WC	7.65	0.66	0.011278	0.9887
UWC	10.62	4.09	0.036264	0.9857
EC	13.05	3.70	0.021726	0.9941
UEC	13.35	5.85	0.032923	0.9901
DES/ChCl-EG	35.08	7.8	0.006338	0.996
UDES/ChCl-EG	39.21	10.70	0.00696	0.995

### 2.8 Peps composition

The phenolic profile of PEPs was examined using high performance liquid chromatography (HPLC, Agilent 1260, Germany). The temperature of column was maintained at 30 °C, and an injection volume was set at 10  μL. The peak areas were measured using external standard method under gradient elusion. The mobile phase of HPLC consisted of solvent A (water-glacial acetic acid, 98:2, v/v) and solvent B (acetonitrile) at a flow rate of 1.0  mL/min. The gradient program was as follows: 0–2  min, 3–15% B; 2–12  min, 15–30% B; 12–25  min, 30% B; 25–27  min, 30–3% B.

### 2.9 *In-vitro* antioxidant activity of PEPs

DPPH assay was conducted by modified method of Mehmood et al. [19]. A 0.1  mM DPPH solution was prepared in methanol and stored at 4 °C in the dark. PEP extracts were obtained with different solvents, with or without UAE (WC, EC, DES, UWC, UEC, and optimized UDES), and diluted to fall within the linear absorbance range. For the assay, 180  µL of sample or Trolox standard was added to 20  µL DPPH in a 96-well plate, with blanks and controls prepared accordingly. After 30  min incubation at 25 ± 1 °C in the dark, absorbance was subsequently measured at 517  nm.

Similarly, ABTS assay was performed according to the Mehmood et al. [19] with minor modifications. A 7.4  mM ABTS stock solution in water was reacted with 2.6  mM potassium persulfate (K_2_S_2_O_8_) to generate ABTS•^+^, which was then stored for 12 h in the dark at room temperature. The solution was diluted with ethanol to achieve an absorbance of 0.70 ± 0.02 at 734  nm. PEP extracts were used as samples. In a 96-well plate, 20  µL of sample or Trolox standard was combined with 180  µL of ABTS•^+^ solution, including appropriate blanks and controls. After 6  min incubation in the dark at 25 ± 1 °C, absorbance was measured at 734  nm.

The reducing power of PEPs was assessed using a modified FRAP assay [18]. Fresh FRAP reagent was prepared by mixing 10  mM TPTZ in 40  mM HCl, 20  mM FeCl_3_·6H_2_O, and 300  mM acetate buffer (pH 3.6) in a 1:1:10 (v/v/v) ratio. PEP was diluted in distilled water to ensure absorbance within the linear range. In a 96-well plate, 20  µL of sample solution or standard of FeSO_4_·7H_2_O was combined with 180  µL of FRAP reagent, including sample and reagent blanks. The absorbance was measured at 593  nm after 4  min incubation at 25 ± 1 °C in the dark.

### 2.10 Structural analysis of PEPs extracts by FTIR

The functional groups of each of the extracted PEPs extracted (UWC, UEC and UDES) were analyzed via FTIR spectroscopy to evaluate the conservancy of key phenolic structural features after extraction with UAE. The PEPs were directly placed on the FTIR sample holder and scanned from 500-4000 cm^–1^ as mentioned in section 2.3 above. The retention of the major absorption bands was used not as a means of determining the quantitative status of the compounds but rather as an indication of the chemical integrity of the compounds.

### 2.11 Observation of PEPs microstructure

The microstructure of PEPs extracted powder by WC, UWC, EC, UEC, DES and UDES was analyzed by scanning electron microscopy (SEM; ZEISS Sigma 300, Germany), following the method of Sun et al. [18] with slight modifications with control sample (*P. eryngii* powder without any changes). The samples were initially washed three times, alternately using anhydrous ethanol and distilled water, followed by freeze-drying to eliminate all moisture. The samples after drying were mounted on aluminum stubs and sputter-coated with a thin gold layer, and examined at 4.5kx magnification with scale bars of 5 µm.

### 2.12 Antifungal activity of PEPs against Aspergillus niger

The antifungal activity of PEPs against *A. niger,* was evaluated using a modified method described by Yang and Jiang [21], applying the agar well diffusion technique [22]. Infected apples tissues exhibiting characteristic brown-black concentric lesions were surface-sterilized using 1% sodium hypochlorite (NaOCl) for 1 min and then three times rinsed with sterile distilled water. Tissue fragments (5 × 5 mm) from lesion margins were diluted tenfold, and 0.1 mL aseptically transferred to potato dextrose agar (PDA) plates and incubated at 28 ± 1 °C for 3–5 days. Pure cultures were obtained by subculturing hyphal tips on fresh PDA. PEP extracts (UDES, UEC, and UWC) were dissolved in sterile water at concentrations of 0, 0.05, 0.1, 0.15, 0.2, 0.25, 0.3, and 0.35 mg/mL and filter-sterilized (0.22 μm). After inoculating the fungal plugs, wells (∼5 mm) were punched in the agar and filled with PEP extracts at different concentrations. Plates were incubated at 28 ± 1 °C for 96 h, and the radial growth of the fungus was measured to determine antifungal activity. The antifungal activity was calculated using Equation (5):

(5) $$\mathrm{Inhibition}\left(\%\right)=\left(\frac{R_{C}-R_{t}}{R_{C}}\right)\times 100$$

where *R_c_* is the radial growth (cm) of *A. niger* in control plates and *R_t_* is the radial growth in PEP-treated plates.

### 2.13 Statistical analysis

All experiments were carried out in triplicate, and results are presented as mean ± standard deviation (SD). Statistical analysis was performed using SPSS (IBM SPSS Statistics 26). Differences among group means were evaluated by one-way analysis of variance (ANOVA), followed by Tukey’s post-hoc test, with *P* < 0.05 considered statistically significant. Graphical representations were prepared using OriginPro 2022 (OriginLab, Northampton, MA, USA).

## 3 Results and discussion

### 3.1 Physicochemical properties of DESs

All twelve DESs formed clear, homogeneous, viscous liquids without crystallization or phase separation after equilibration (Table 1), indicating stable interactions between HBAs and HBDs through hydrogen bonding. This observation is consistent with previous studies highlighting the key role of hydrogen bonding in maintaining the structural integrity and homogeneity of DES systems [18].

The physicochemical characterization of the prepared DESs shows a clear temperature-dependent behavior in their viscosities as can be seen in Table 1. In general, those DES prepared using a polyol; such as EG, have a lower viscosity than those DES prepared using a HBD in the form of either an organic acid or sugar. When compared with increasing temperature, the range of viscosities exhibited a decreasing trend relative to increasing temperature, which is primarily linked to decreased hydrogen bonding at thermal extremes [23]. At low temperatures, the stronger intermolecular hydrogen bonding restricts the mobility of DES components which produces a higher viscosity. As the temperature increases, the intermolecular hydrogen bonding weakens, allowing greater molecular mobility and consequently reducing viscosity. Water dilution (10–20%, v/v) caused a significant decrease in viscosity caused by the partial destruction of the hydrogen bonding network and increasing fluidity. In regards to the polyol-based DES, the smaller molecular size and weaker hydrogen bonding further contributed to lower viscosities, highlighting the strong influence of hydrogen bonding on the rheology of these systems [23], [24].

As shown in Table 1, the densities of the synthesized DESs varied from 0.77 g/cm^3^ for Be-MA at 70 °C to 1.39 g/cm^3^ for Glu-CA at 20 °C, all being significantly higher than the density of water. The decrease of densities with rising temperature is consistent with previous findings [24], attributed to the length of the carbon chain of the HBD in relation to the hydrogen bond interactions within the solvent system. Correspondingly, the acidity and alkalinity of the DESs were also influenced by the intrinsic properties of the selected HBDs (Table 1) [25]. The synthesized DESs exhibited broad pH range from about 0.9 (most acidic) to 9.6 (most basic). Acidic DESs had an acidic pH value, whereas polyol-derived DESs were closer to neutral. This tunable pH of DES is particularly advantageous for targeted extraction applications, as pH significantly affects the solubility and stability of bioactive compounds. Moreover, variations in temperature were observed to affect the physicochemical properties of DESs. As previously discussed, increasing temperature weakens the hydrogen bonding and ion–dipole interactions, thus influencing the acid/base characteristics of the entire system [26]. This highlights the dual role of component selection and temperature in tailoring the physicochemical behavior of DESs.

**Fig. 1:**
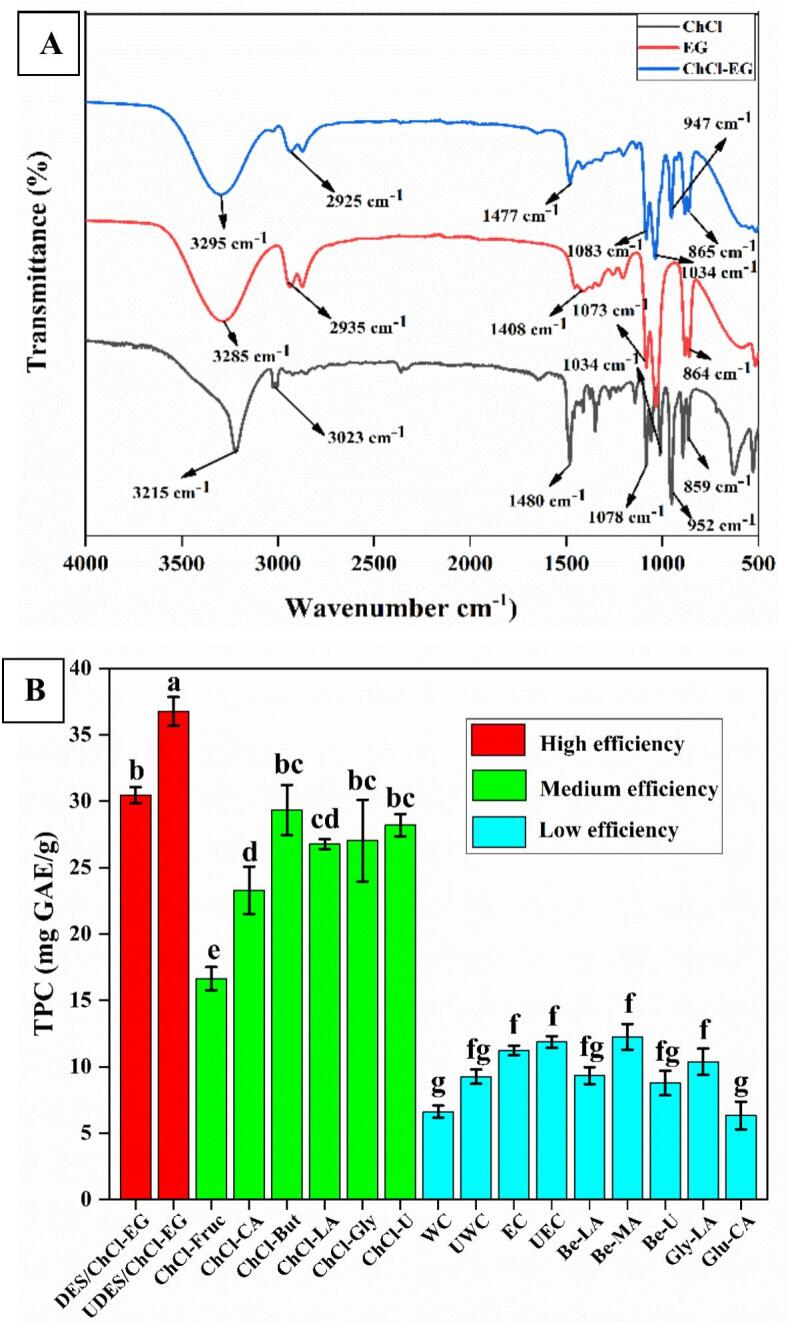
**(A)** Comparative FTIR spectra of choline chloride (ChCl), ethylene glycol (EG), and their DES combination (ChCl-EG) showing hydrogen bonding interactions. **(B)** Effect of different extraction solvents on the yield of PEPs obtained using UAE and conventional solvent with and without UAE extraction methods.

FTIR spectra of ChCl-EG (1:2), which exhibited the highest PEPs yield, exposed the characteristic hydrogen-bonding interactions between HBA and HBD components (Fig. 1A). Spectra from the individual ChCl and EG components as well as optimized DES ChCl-EG were observed and all showed a shift in their respective characteristic peaks as evidence of a new hydrogen bond being formed between the ChCl and EG components. The distinct absorption bands observed in the O-H stretching region (3600–2800 cm^–1^) provides further confirmation that hydroxyl groups are participating in hydrogen bonding of the DES. Finally, the absence new peaks in the DES spectrum indicates that hydrogen bonding is the predominant stabilizing interactions within the eutectic mixture of ChCl and EG and that there were no new covalent bonds formed between the two components [24], [27]. The broad O-H stretching band of pure EG at 3285 cm^–1^ shifted to 3295 cm^–1^ in the DES, with notable peak broadening, signifying enhanced hydrogen bonding between the chloride anion and hydroxyl groups. Similarly, the C-O stretching vibration, observed at 1073 cm^–1^ in EG and 1078 cm^–1^ in ChCl, shifted to 1083 cm^–1^ in the DES, reflecting weakened C-O bonds due to chloride-mediated interactions. In addition, the merging of C-H stretching vibrations (2925–3023 cm^–1^) pointed to alterations in the local electronic environment. The disappearance of the free O-H band near 3500 cm^–1^, together with broadening in CH_2_ bending (1477 cm^–1^) and C-Cl stretching (865–952 cm^–1^) modes, further confirmed the establishment of a dense hydrogen-bonding network within the DES. These structural modifications account for the superior efficiency of the ChCl-EG (1:2) DES in PEPs extraction.

### 3.2 Screening and selection of the optimal DES for PEP extraction

The 12 synthesized DESs, along with WC and EC, showed substantial differences in PEP extraction efficiency, mainly due to the variations in viscosity, density, pH, and hydrogen bonding capacity among the DES systems. As shown in Fig. 1B, DES of ChCl-EG exhibited the maximum extraction yield (30.46 mg GAE/g), whereas Glu-CA showed the lowest (6.33 mg GAE/g). Water also displayed relatively poor performance (6.62 mg GAE/g), yielding less than ethanol and most DESs. The differences in extraction efficiency are strongly linked to solvent viscosity, which is primarily determined by the hydrogen bonding interactions between HBAs and HBDs [28]. ChCl-EG displayed a relatively low viscosity (110 mPa·s) and correspondingly achieved the highest PEPs extraction. In contrast, Glu-CA exhibited the highest viscosity (745 mPa·s) and the lowest yield at 45 °C, indicating that high-viscosity DESs hinder polyphenol diffusion and solubilization (Table 1). These findings supports the previously established principle that the viscosity of a liquid has an effect on the cavitation performance and mass transfer that occurs when using ultrasonication [28]. Similarly, Sun et al. [18] demonstrated that the water addition reduces viscosity of DES, and thus improves its ability to dissolve substances leading to a higher yield of polyphenol being extracted from grape seeds. Correspondingly, DESs with lower viscosities such as ChCl-EG, ChCl-U, ChCl-Gly and ChCl-LA promoted greater mobility of PEP molecules and improved diffusion rates, ultimately leading to higher extraction yields. Based on the observed relationship between viscosity and extraction efficiency (Fig. 1B), the 12 DESs were broadly categorized into three groups: high, medium, and low efficiency. High-viscosity systems, such as Glu-CA, were consistently unfavorable for PEP extraction, whereas low-viscosity DESs, particularly ChCl-EG and ChCl-But, achieved superior yields. Other ChCl-based DESs showed moderate performance, ranging from 16.64 ± 0.87 mg GAE/g (ChCl-Fruc) to 29.34 ± 1.89 mg GAE/g (ChCl-But). In contrast, betaine-based DESs exhibited lower efficiency (8.77 ± 0.92 to 12.26 ± 0.96 mg GAE/g), while Glu-CA was the least effective (6.34 ± 1.04 mg GAE/g), likely due to Maillard reaction interference during extraction [29]. Accordingly, ChCl-EG was selected as the optimal extraction solvent for subsequent experiments.

Moreover, the optimized ChCl-EG system with UAE (UDES/ChCl-EG) exhibited the highest polyphenol yield (36.77 ± 1.07  mg GAE/g) compared to without UAE (DES/ChCl-EG) showing 30.46 ± 0.60  mg GAE/g. Similarly, WC and EC increasing from 6.62 to 9.26  mg GAE/g and from 11.23 to 11.86  mg GAE/g with UAE, respectively. The increased polyphenol yield with UAE confirms the positive effect of ultrasonication on the extraction efficiency, consistent with the previous reports [30]. Ultrasonication significantly enhanced PEPs recovery across all tested systems compared with simple heat-assisted extraction. The combined application of DESs and ultrasonication thus provides a highly effective strategy for enhancing the extraction of PEPs.

### 3.3 Influence of extraction parameters on PEPs yield

**Fig. 2:**
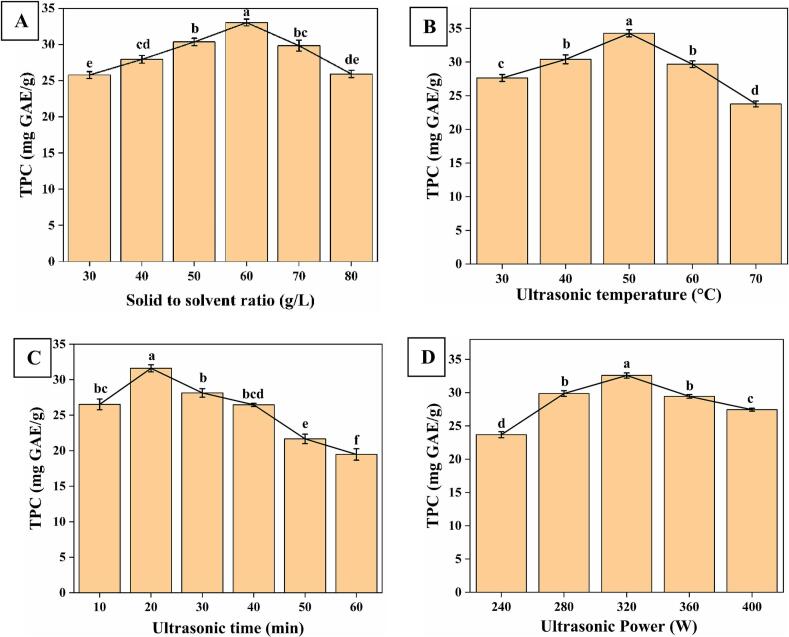
Effects of different extraction conditions on the yield of PEPs: **(A)** Solid-to-solvent ratio **(B)** Ultrasonication temperature, **(C)** Ultrasonication time, and **(D)** Ultrasonication power. Note: PEPs (*Pleurotus eryngii* polyphenols); GAE (gallic acid equivalent); TPC (Total Phenolic Content). Different letters indicate significant differences between treatments (*P < 0.01*)*.*

The extraction yield of PEPs was significantly affected by process parameters, including solid-to-solvent ratio, ultrasonication temperature, ultrasonication time, and ultrasonication power. As shown in Fig. 2A, the extraction yield of PEPs increased with increasing solid-to-solvent ratio from 30 to 60  g/L, reaching a maximum yield of 33.2 mg GAE/g, followed by gradual declined at higher ratios. Statistical analysis confirmed significant differences among the tested ratios (*P < 0.05*). The optimal extraction ratio for PEP was found to be 60 g/L, balancing extraction efficiency with commercial feasibility. The improved extraction efficiency at moderate ratio may be likely due to increased mass transfer driven resulting from the concentration gradient between intracellular and extracellular regions. However, ratios below 60  g/L led to underutilization of the solvent capacity, whereas higher ratios condensed extraction efficiency, probably due to solute saturation or dilution effects. Recent studies demonstrated that excessive loading of solid leads to decreased solvent flow due to increased viscosity, and polyphenol-polyphenol interactions that promote re-aggregation [31]. In addition, high solid loading could negatively affect cavitation efficiency in ultrasound treatment since it reduces bubble formation and collapse, thus minimizing mass transfer efficiency and cell wall disruption [32]. Similar findings haves been reported in previous studies on polyphenol extraction from mushrooms, where a high solid loading was seen to limit phenolic content recovery as a result of limited solvent diffusion into the cells [33]. These results showed that efficient PEP extraction depends on maximizing solvent penetration and polyphenol solubility, highlighting the need to optimize the solid-to-solvent ratio to achieve high yields while minimizing solvent consumption.

Simultaneously, the effect of ultrasonic temperature on PEPs is explained in Fig. 2B. As the temperature increased from 30 °C to 50 °C, PEPs yield rose from 27.6.0 ± 0.5 to 34.27 ± 0.53, reaching a maximum at 50 °C, showing as an optimum temperature. Further elevation to 60 °C and 70 °C resulted in a progressive decline to 29.7 ± 0.49 and 23.8 ± 0.43 mg GAE/g, respectively. A similar trend was observed by Sun et al. [18], who reported the highest TPC (118.5 mg GAE/g) from *Cabernet Sauvignon* seeds at an optimum temperature of 50 °C. When the temperatures was below 50 °C, increased thermal energy improved extraction through decreased DES viscosity and surface tension, which improving solvent diffusion into the cellular matrix, and increasing polyphenol solubility [34]. Statistical analysis revealed significant differences in the extraction yields across the tested temperature range (*P < 0.05*). As the temperature rises above 50 °C, the extraction efficiency graphs showing declining behavior. While moderate heating improved mass transfer, high temperatures induced oxidative degradation and structural variations of polyphenols, thereby decreasing yield [35]. Moreover, the higher temperatures reduce solvent viscosity as well as surface tension, and facilitating bubble collapse, release energy, thereby potentially fragmenting the molecular structure of the solute [36], [37]. Therefore, the optimum ultrasonication temperature 50 °C is a good balance between achieving the maximum yield of extracted polyphenols and limiting the thermal degradation of sensitive components.

Ultrasonication time greatly affects phenolic extract yields, where the maximum yield occurred at 20 min (31.60 mg GAE/g) (Fig. 2C). Increasing the duration of the ultrasonication process promotes cavitation phenomenon, enhances the mass transfer, and allows for greater solubility of polyphenols and therefore higher extraction rates [28]. However, after reaching a maximum extraction rate at the optimal time (20 min), yields are shown to decline gradually (28.14 mg GAE/g at 30 min; 26.46 mg GAE/g at 40 min). Finally, by 60 min yields drop significantly (19.48 mg GAE/g), possibly due to polyphenol degradation through oxidative reactions as a result of long exposure to ultrasonic waves, as well as through free radical formation and polymerization of the polyphenols themselves [18]. The results of the statistical analysis indicate that significant differences exist among the extraction times evaluated (P < 0.05).

The extraction yield of PEPs was increased by increasing ultrasonication power, reaching a maximum of 32.58  mg GAE/g at 320  W (Fig. 2D). The high yield of TPC at 320 W indicates that the stronger cavitation effects at moderate power facilitate solvents to penetrate more easily into the fungus, thereby disrupting the cell wall and increasing the movement of intracellular compounds [30]. At higher powers (360 W and 400 W), the extraction yield decreased to 29.43 mg GAE/g and 27.43 mg GAE/g, respectively, due to possible oxidative or structural damage to the phenolic compounds, which was also seen with the extraction of flavonoids from *Amorpha fruticosa* leaves [38]. Thus, it was determined that 320 W was the optimal power for ultrasonication to extract the maximum yield of PEPs.

### 3.4 Optimization of the PEPs extraction parameters by RSM

**Fig. 3:**
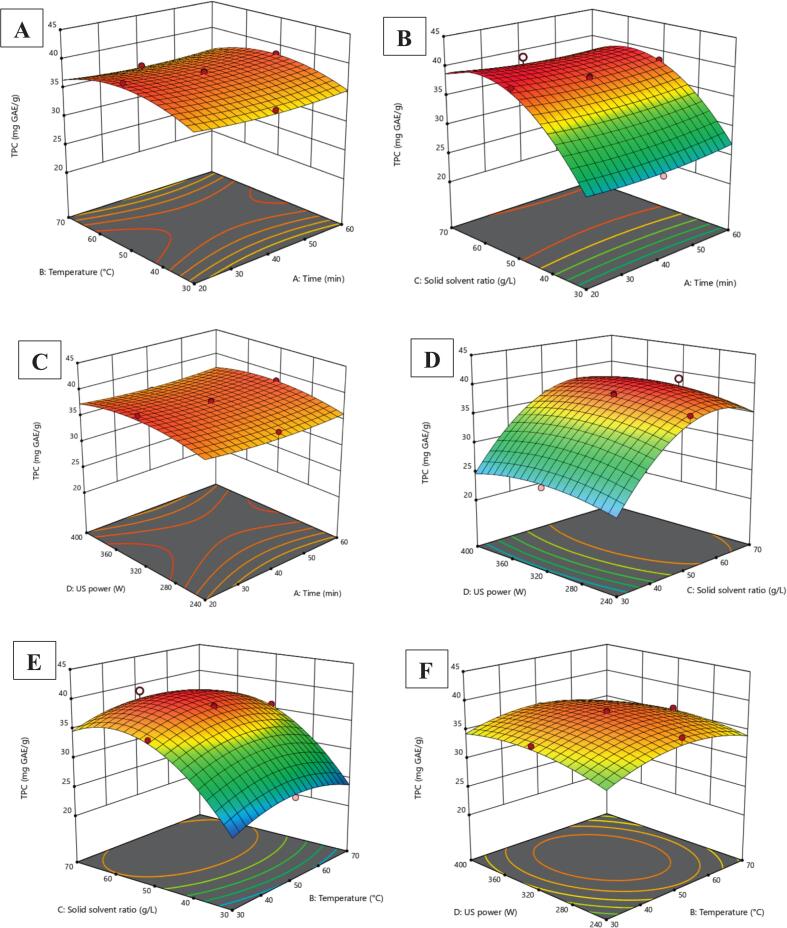
Response surface plots (**A–F**) illustrating the interactive effects of ultrasonication time **(A)**, ultrasonication temperature **(B)**, solid-to-solvent ratio **(C)**, and ultrasonication power **(D)** on the extraction yield of PEPs. Note: PEPs (*Pleurotus eryngii* polyphenols); GAE (gallic acid equivalents); TPC (Total Phenol Content).

As shown in Fig. 3, RSM revealed that ultrasonication time (A), temperature (B), solid-to-solvent ratio (C), and power (D) significantly influenced PEP yield. The model established a strong correlation between theses experimental variables and extraction efficiency, with total PEP yield (Y) serving as the response parameter. The fitted regression equation (6) quantitatively describes these relationships and was subsequently used to identify optimal extraction conditions.

(6) $$\mathrm{TPC}/Y=-62.6204+1.85069C-0.00121359\mathrm{AC}-0.000351711\mathrm{BD}-0.00645962B^{2}-0.0154177C^{2}-0.000259437D^{2}$$

ANOVA for the regression model indicated that the model was highly significant (*P < 0.001*), while the lack-of-fit term was not significant (P = 0.0663 > 0.01), confirming the suitability of the model (Table 5). The coefficient of determination (R^2^ = 0.9903) further demonstrated excellent agreement between the predicted and experimental data. Among the factors, the effect on extraction efficiency decreased in the order: C > D > B > A. Significant terms included the linear coefficient of C, quadratic coefficients B^2^, C^2^, D^2^, and interaction terms AC and BD (*P < 0.05*). The three-dimensional response surface and contour plots illustrate the interactions between variables on PEPs extraction (Fig. 3). Using the regression model (Table 5), the predicted optimal TPC was 39.601 mg GAE/g, corresponding to an ultrasonication time of 27 min, power of 351.04 W, temperature of 49.23 °C, and solid-to-solvent ratio of 58 g/L.

**Table 5:** ANOVA for the regression model of PEPs extraction yield.

**Source**	**Sum of Squares**	***df***	**Mean Square**	***F*-value**	**Regression Coefficient**	***p*-value**	**Significant level**
**Model**	1274.22	14	91.02	130.97	Intercept = 37.91	< 0.0001	significant
A-Time	0.7198	1	0.7198	1.04	−0.2000	0.3223	
B-Temperature	0.9950	1	0.9950	1.43	0.2351	0.2470	
C-S/S/R	575.73	1	575.73	828.43	5.66	< 0.0001	
D-US power	2.85	1	2.85	4.10	0.3978	0.0580	
AB	9.025E-07	1	9.025E-07	1.299E-06	0.0002	0.9991	
AC	3.77	1	3.77	5.43	−0.4854	0.0317	
AD	0.2378	1	0.2378	0.3421	0.1219	0.5659	
BC	0.0134	1	0.0134	0.0193	−0.0290	0.8910	
BD	5.07	1	5.07	7.29	−0.5627	0.0146	
CD	0.2710	1	0.2710	0.3899	0.1301	0.5402	
A^2^	1.11	1	1.11	1.59	0.6520	0.2232	
B^2^	17.37	1	17.37	24.99	−2.58	< 0.0001	
C^2^	98.95	1	98.95	142.39	−6.17	< 0.0001	
D^2^	7.17	1	7.17	10.32	−1.66	0.0048	
**Residual**	12.51	18	0.6950				
**Lack of Fit**	9.88	10	0.9878	3.00		0.0663	not significant
Pure Error	2.63	8	0.3289				
**Cor Total**	1286.73	32					
**R^2^**	**0.9903**						
Adjusted R^2^	0.9827						
Predicted R^2^	0.9556						
Std. Dev.	0.8336						
Mean	32.58						
C.V. %	2.56						

Under these conditions, the experimentally determined TPC was 37.028 ± 1.7 mg GAE/g, closely matching the predicted value with a relative error of only 3.47% (Table 6). These results validate the RSM model and confirm the effectiveness of the optimized UDES extraction conditions for maximizing PEPs yield.

**Table 6:** Experimental and predicted values of response at optimum conditions.

**Optimum conditions**	**Coded levels**	**Actual levels**
Time (min)	−0.6497	27.006
Temperature (°C)	−0.0385	49.23
Solid to solvent ratio (g/L)	0.4	58
Ultrasonic power (W)	0.338	351.04
**Response**	**Predicted value**	**Experimental value**
TPC (mg GAE/g)	39.601	37.028

### 3.5 Extraction kinetics study

**Fig. 4:**
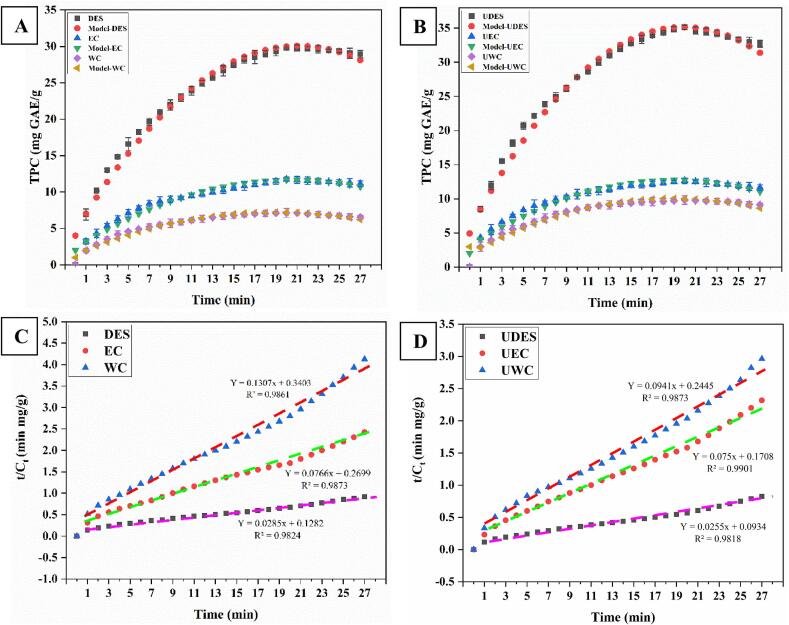
**(A)** Comparative extraction process of PEPs using different solvent extracts without UAE and **(B)** with UAE; **(C)** together with the fitted second-order kinetic curves for different solvent extracts without UAE and **(D)** with UAE. Note: DES (deep eutectic solvent); EC (ethanol control); WC (water control); UDES (Ultrasonicated deep eutectic solvent), UWC (ultrasonicated water control), and UEC (ultrasonicated ethanol control) and PEP (*Pleurotus eryngii* polyphenols).

Fig. 4 represents the extraction performance and kinetic modeling of PEPs under different solvent systems. In the absence of UAE, the optimized DES exhibited higher extraction efficiency than both EC and WC, with maximum yield of approximately 29.80 mg GAE/g. In contrast, EC and WC showed lower extraction capacities, achieving approximately 11.75 mg GAE/g and 7.14 mg GAE/g, respectively (Fig. 4A). According to the present findings, UDES achieved a maximum PEP yield exceeding 35.11 mg GAE/g, which was higher than that obtained with the non-ultrasonicated DES system. Similarly, UEC and UWC also showed improved performance compared to their respective controls (Fig. 4B). These results indicate that UAE enhance the intrinsic extraction capability of the DES, and benefitting the most from this combined effect. This observation is consistent with the previous findings [39].

The extraction kinetics of PEPs were well described by the second-order kinetic model showing strong correlation (R^2^ > 0.98) (Fig. 4C&D). As showing in table 4, DES (ChCl-EG) exhibited the highest equilibrium concentration, which was further enhanced by UAE. These results demonstrate the strong extraction capability of DES and the effectiveness of ultrasound in improving extraction efficiency (UDES). The WC and EC systems had lower values for C*_s_* as compared to UEC and UWC, which resulted in higher rate constants (*k*) for UEC and UWC due to the enhanced mass transfer caused by ultrasound. Interestingly, while DES had lower *k* values than both EC and WC, the greater solubilization capacity of DES produced higher values for initial extraction rate (*h*) and thus the overall best performing DES/UAE system (UDES). These results demonstrate that the use of DES in combination with UAE not only increases the amount extracted, but also improves rate performance compared to traditional solvents. Therefore, DES has potential to be utilized as an efficient and sustainable solvent for extracting PEPs. Further enhancement of kinetics and yields can be accomplished by using UAE, thus making UDES the optimal method for maximizing recovery.

### 3.6 Antioxidant properties of PEPs extracted with different solvents

**Fig. 5:**
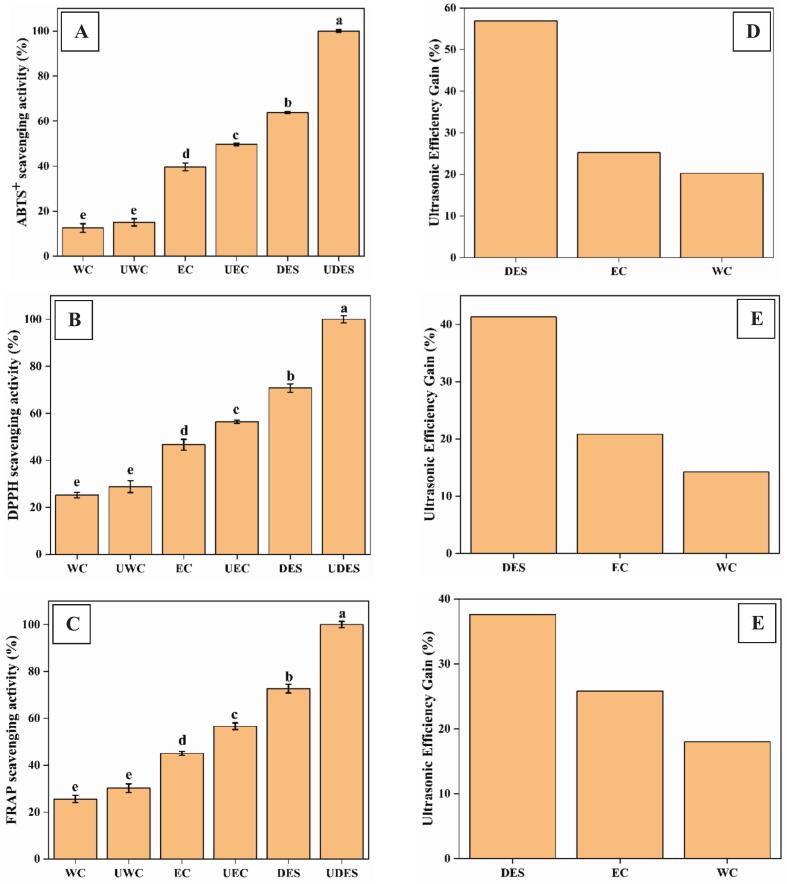
Antioxidant activity of PEPs extracted from different solvent system with and without UAE **(A-C)**. Enhancement ratio of antioxidant capacity of PEPs by UAE under different solvent systems **(D-F).** Note: PEPs (*Pleurotus eryngii* polyphenols); UAE (ultrasound-assisted extraction). Different letters indicate significant differences between groups (*P < 0.01*).

Polyphenols, are the secondary metabolites of plants and fungi, often produced in greater amounts under environmental stress conditions, where they contribute to defense mechanisms and adaptive resilience. As illustrated in Fig. 5A-C, the type of solvent obviously affected the antioxidant activity of the extracts. The PEPs produced with optimized DES exhibited the highest capacity for scavenging free radicals as evidenced by the ABTS^+^, DPPH•, and FRAP assay results, which were all normalized to 100%. In contrast, water proved to be the least efficient solvent, yielding values of only 12.50%, 25.23%, and 25.59% for ABTS^+^, DPPH•, and FRAP, respectively. The polarity and hydrogen-bonding capacity of DES surpass those of conventional solvents, leading to higher solubilization of polyphenolic compounds. Indeed, DESs are able to form extensive hydrogen bonds with phenolics, thereby improving their stability and extraction yield [40]. In agreement with Nam et al. [41], certain DES components such as L-proline may also act synergistically with natural compounds, further enhancing the antioxidant potential of extracts.

UAE markedly improved antioxidant activities for WC, EC, and DES (Fig. 5D–F) relative to without UAE. Specifically, ABTS^+^ activity with UAE increased by 56.88%, 25.26%, and 20.25% in DES, EC and WC, respectively, while DPPH• activity and FRAP activity also were improved by ultrasonication conditions. These results are consisted with previous findings which show that ultrasonication enhances polyphenol recovery by disrupting cell walls, indirectly increasing antioxidant activity through improved extraction of bioactive compounds [39].

### 3.7 Influence of extraction solvents on the functional groups of PEPs

**Fig. 6:**
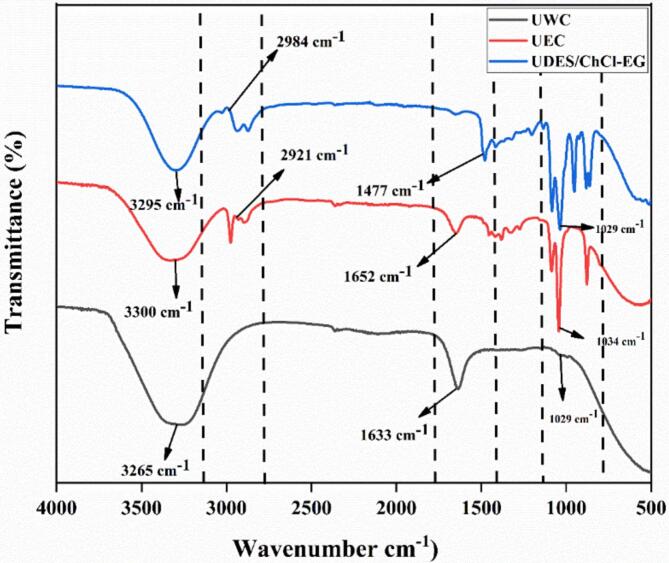
Comparison of FTIR spectra revealing functional group interactions in PEPs extracted with different solvents. Note: UDES/ChCl-EG (Ultrasonicated deep eutectic solvent/choline chloride-ethylene glycol), UWC (ultrasonicated water control), and UEC (ultrasonicated ethanol control).

The FTIR analysis showed notable differences in functional group interactions among PEPs extracted using UDES/ChCl-EG, UWC, and UEC solvents (Fig. 6). One of the largest differences was evident in the O-H stretching range between 3180–2840 cm^–1^ where, PEP from UDES/ChCl-EG had a notable peak shift at 3295 cm^–1^ compared to UWC at 3265 cm^–1^ and UEC at 3300 cm^–1^. The downward movement of the peak indicates an increased amount of hydrogen bonding occurring between PEP and the UDES solvent system, which is in line with earlier reports showing enhanced stabilization due to hydroxyl interactions [42].

All extract spectra exhibited a predominant set of peaks around 2921 and 2984 cm^−1^ (C-H stretching), 1652 and 1633 cm^−1^ (C=O stretching), and 1477 cm^−1^ (aromatic C=C bending). This indicates that the primary structure of polyphenols from a given source has remained intact through all methods of extraction. However, the UDES-extracted PEPs demonstrated additional peak broadening at 1633 cm^–1^, which is commonly attributed to heterogeneous hydrogen bonding interactions involving carbonyl groups, consistent with interactions between PEPs and the hydroxyl rich ChCl–EG system. This results of current study aligns with previous findings [18], [43], that demonstrated similar types of interactions between UDES and polyphenols using FTIR. Comparative analysis of the fingerprint region (1500–900 cm^–1^) exposed minor but consistent shifts in UDES/ChCl-EG extracts, particularly near 1150 cm^–1^ (C-O stretching) and 900 cm^–1^ (aromatic C-H bending). These variations suggest the formation of new hydrogen-bonding interaction between PEPs and UDES components, which likely contribute to the improved extraction efficiency and stability observed in our study. Overall, these results indicate that while all solvents maintain basic polyphenol functionality, UDES systems promote specific molecular interactions that improve both extraction yield and compound stabilization through tailored hydrogen bonding networks.

### 3.8 HPLC profile of PEPs

**Fig. 7:**
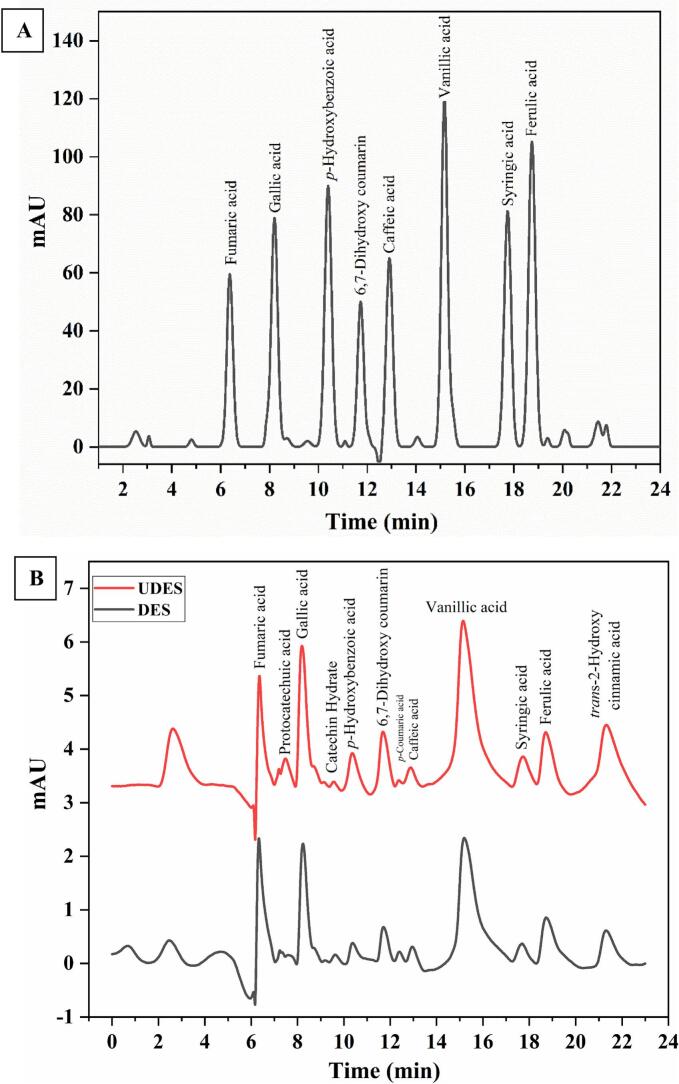
HPLC analysis of PEPs. **(A)** Chromatograms of phenolic compound standards used for peak identification in PEPs. **(B)** HPLC chromatograms of polyphenols extracted using the optimized DES with and without UAE, showing differences in peak intensity and profile. Note: DES (deep eutectic solvent extraction without UAE); UDES (deep eutectic solvent extraction with UAE); PEPs (*Pleurotus eryngii* polyphenols).

The HPLC chromatogram of PEPs demonstrates the presence of several peaks corresponding to characteristic phenolic compounds obtained using DES system (Fig. 7A&B). A total of 12 distinct polyphenolic compounds has been identified through this study, including: fumaric acid, protocatechuic acid, gallic acid, catechin hydrate, *p*-hydroxybenzoic acid, 6,7-dihydroxy coumarin, *p*-coumaric acid, caffeic acid, vanillic acid, syringic acid, ferulic acid, and *trans*-2-hydroxycinnamic acid. Among these fumaric acid, 6,7-dihydroxy coumarin, and caffeic acid were detected for the first time as being produced from *P. eryngii* production waste, providing further evidence of the efficacy of UDES method to extract the bound phenolics. Eight of these 12 compounds have been verified via comparison with their corresponding authentic standard compounds, while the other four have been tentatively identified based on their respective retention times and UV spectra when compared with literature values [12], [44]. Among these compounds, vanillic acid and gallic acid were identified as the major phenolic acids found in PEPs, showing the highest peak intensities and concentrations of about 9.14 mg/g and 7.46 mg/g, respectively (Fig. 7B). Conspicuously, the chromatogram of UDES exhibited significantly higher peak intensities than that of DES extraction, indicating enhanced cell wall disruption and mass transfer tempted by ultrasonic cavitation, which facilitated the release and solubilization of intracellular polyphenols [28]. The major increases in fumaric acid, 6,7-dihydroxy coumarin, ferulic acid, and syringic acid observed in the UDES chromatograms suggest that high energy ultrasonication can effectively liberate bound phenolic acids which are commonly associated with the cell walls of mushrooms. The enhanced profile observed with UDES is consistent with previous findings by Sun et al. [18], who demonstrated that catechin and epicatechin were main components in UDES-extracted from grape seed polyphenols. The wide variety and range of phenolic acids present within the PEPs indicate the potential of this phenolic acid profile to possess strong antioxidant potential due to their unique chemical structure.

### 3.9 SEM observation of microstructural changes in *p. Eryngii* powder during PEP extraction

**Fig. 8:**
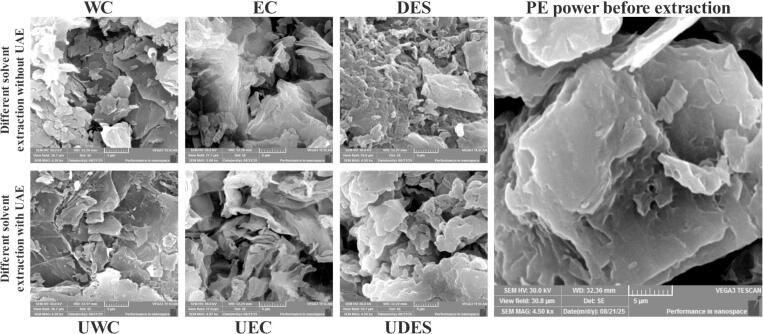
Microstructure of *P. eryngii* powder before extraction (PE powder) and after extraction using different solvents, with or without UAE examined at 4.5kx magnification with scale bars of 5 µm. Upper row: extraction without UAE using WC, EC, and DES. Lower row: extraction with UAE using UWC, UEC, UDES. Note: UAE (ultrasonic-assisted extraction); WC (water control); EC (ethanol control); UWC (water control with UAE); UEC (ethanol control with UAE); and PEPs (*Pleurotus eryngii* polyphenols).

SEM analysis exposed a clear structural difference in *P. eryngii* before and after extraction of PEPs from the mushroom powder (Fig. 8). The surface of the raw *P. eryngii* powder (PE powder) prior to extraction appeared relatively smooth, flat, and dense with intact cellular structures. This compact morphology act as a significant physical barrier, limiting solvent diffusion and the subsequent release of intracellular compounds such as PEPs. Substantial microstructural changes were observed in all samples after extraction; the most pronounced alteration associated with the application of UAE. The extractions without UAE (WC, EC, and DES) exhibited moderate surface erosion and partial disruption of the dense structure. This suggests that the chemical action of the solvents alone, including that of DES/ChCl-EG, can gradually solubilize and partially degrade the cell wall components over time.

In contrast, the UAE-treated samples (UWC, UEC, and UDES) showed severe and extensive structural disruption. Their surfaces became highly fractured, and porous, with a distinct honeycomb-like morphology. This pronounced transformation is a direct consequence of the ultrasonic cavitation effect (Fig. 8). Ultrasonic waves promote the rapid formation, growth, and collapse of microscopic cavitation bubbles, generating localized high temperature, pressure, and shear forces along with the erosive action of DES. These combined effects produce numerous microchannels and pores within the plant matrix, as reflected by the honeycomb-like structure in UAE-treated samples [45]. A similar pattern of microstructural disruption was observed in *P. eryngii* powder, where UDES treatment transformed the surface from a compact to a fractured, porous morphology. This observation is agrees with Sun et al. [18], who reported similar transitions in grape seeds subjected to UDES treatment. Overall, these findings indicate that the combined action of UAE and DES effectively enhances polyphenol extraction efficiency, explaining the significantly higher yields obtained with UAE, particularly using the optimized UDES system.

### 3.10 Influence of different extraction methods on the antifungal activity of PEPs

**Fig. 9:**
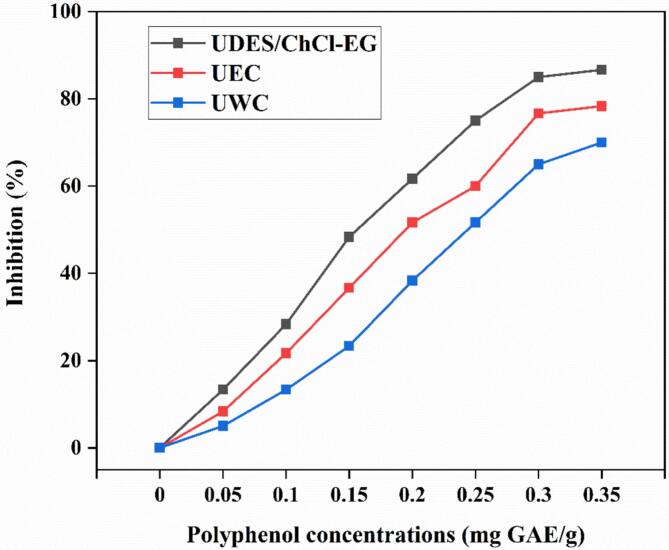
Comparison of inhibitory activities of PEPs extracted by different methods against *A. niger*. Note: UDES/ChCl-EG (Deep eutectic solvent of Choline chloride-Ethylene glycol), UEC polyphenols extracted through ethanol solvent assisted with ultrasonication) and UWC (polyphenols extracted through water as a solvent assisted with ultrasonication).

The extraction method significantly influenced the antifungal activity of PEPs. UDES extracts exhibited the greatest antifungal inhibition of 86.6% at a concentration of 0.35 mg/mL, while substantial inhibition (85%) was observed at 0.30 mg/mL (Fig. 9). In comparison, PEPs extracted from UEC and UWC required higher concentrations (≥0.35 mg/mL) to exert similar levels of fungal growth suppression, showing maximum inhibition rates of 78.3% and 70.0%, respectively. The concentration-dependent inhibitory trend clearly demonstrates that UDES had a superior antifungal activity at lower dosages compared to UEC and UWC. This enhanced antifungal performance may be associated with the higher recovery of phenolic acids and other bioactive constituents obtained through the combined effects of ultrasonication and DES extraction. Ultrasonication promotes cell wall disruption and improves mass transfer, while DESs enhance the solubility and stabilization of phenolic compounds, resulting in extracts with greater biological activity [15]. In addition, the improved extraction efficiency of UDES may have contributed to the recovery of a broader spectrum of antifungal metabolites, including phenolic acids and coumarin derivatives, which are known to interfere with fungal cell membrane integrity and cellular metabolism [16], [17]. Likewise, Zhou et al. [46] reported that the stabilizing interactions between UDES and catechins lower their minimum inhibitory concentrations against *Staphylococcus aureus* and *Pseudomonas putrefaciens*. These findings suggest that UDES not only serves as an efficient extraction medium but also enhances the stability and bioactivity of polyphenolic compounds.

The stronger antifungal activity observed in the present study also supports the potential application of UDES-derived PEPs as natural antifungal agents in food preservation and functional food systems. The ability to achieve high antifungal efficacy at relatively low concentrations may provide advantages for reducing the use of synthetic preservatives while improving the stability and safety of food products.

## 4 Conclusion

This current study demonstrated that UDES extraction is a highly efficient and sustainable strategy for recovering bioactive polyphenols from *P. eryngii* processing by-products. Among the synthesized DESs, ChCL-EG showed the highest extraction efficiency, yielding a maximum of 37.028 ± 1.3 mg GAE/g after optimization using RSM, which closely matched the predicted value. Structural analyses confirmed strong hydrogen-bonding interactions and enhanced stability of PEPs under UDES treatment. Vanillic acid and gallic acid were identified as the major phenolic compound, while fumaric acid, 6,7-dihydroxycoumarin, and caffeic acid reported for the first time in *P. eryngii* extract. The identification of these compounds adds to the existing knowledge of the polyphenolic profile of this species and reinforces its potential as a source of bioactive phenolics. Moreover, UDES-extracted PEPs exhibited higher antioxidant and antifungal activities compared with PEPs extracted from conventional water and ethanol-based extraction methods, including strong inhibitory activity against *A. niger*. Overall, UDES represents a green and environmentally friendly extraction strategy that enhance the yield, stability, and bioactivity of mushroom-derived polyphenols. These findings provide a scientific foundation for the valorization of PEPs in nutraceutical, functional food, and natural antifungal applications. Future studies should focus on process scale-up, industrial feasibility, and *in-situ* validation within the food systems to unlock their full potential.

**Data availability**

Data will be made available on upon reasonable request.

## Funding declaration

This research was supported by National Key Research and Development Program for Intergovernmental International Science and Technology Innovation Cooperation (2024YFE0109500), the SanNongJiufang Science and Technology Cooperation Project of Zhejiang Province (2026SNJF022, 2025SNJF007-01), and Ongoing Research Funding program (ORF-2026–1074), King Saud University, Riyadh, Saudi Arabia.

## CRediT authorship contribution statement

**Muhammad Waheed Iqbal:** Writing – original draft, Methodology, Investigation, Formal analysis, Conceptualization. **Ping Shao:** Visualization, Supervision, Project administration, Funding acquisition, Conceptualization. **Yang Lin:** Writing – review & editing, Validation, Supervision, Project administration, Investigation, Funding acquisition. **Sanabil Yaqoob:** Formal analysis, Data curation. **Muhammad Shoaib:** Formal analysis. **Qing Shen:** Writing – review & editing, Validation. **Moneera O. Aljobair:** Writing – review & editing, Funding acquisition. **Isam A. Mohamed Ahmed:** Writing – review & editing, Funding acquisition.

## Declaration of competing interest

The authors declare that they have no competing financial or personal interests that could have affected the outcomes of this study.
